# Stealth Biocompatible Si-Based Nanoparticles for Biomedical Applications

**DOI:** 10.3390/nano7100288

**Published:** 2017-09-23

**Authors:** Wei Liu, Arnaud Chaix, Magali Gary-Bobo, Bernard Angeletti, Armand Masion, Afitz Da Silva, Morgane Daurat, Laure Lichon, Marcel Garcia, Alain Morère, Khaled El Cheikh, Jean-Olivier Durand, Frédérique Cunin, Mélanie Auffan

**Affiliations:** 1 CNRS, IRD, Coll de France, CEREGE, Aix Marseille Université, 13545, Aix en Provence, France; wei.yanzi.liu@gmail.com (W.L.); angeletti@cerege.fr (B.A.); masion@cerege.fr (A.M.); 2 Institut Charles Gerhardt Montpellier, UMR 5253 CNRS-ENSCM-UM, Ecole Nationale Supérieure de Chimie Montpellier, 8 rue de l’Ecole Normale, 34296 Montpellier, France; arnaud.chaix@enscm.fr (A.C.); durand@univ-montp2.fr (J.-O.D.); frederique.cunin@enscm.fr (F.C.); 3 Institut des Biomolécules Max Mousseron, UMR 5247 CNRS-UM, 15 Avenue Charles Flahault, BP 14491, 34093 Montpellier CEDEX 05, France; magali.gary-bobo@inserm.fr (M.G.-B.); afitz@hotmail.fr (A.D.S.); morgane.daurat2@gmail.com (M.D.); laure.lichon@umontpellier.fr (L.L.); marcel.garcia@inserm.fr (M.G.); alain.morere@umontpellier.fr (A.M.); 4 NanoMedSyn, 15 Avenue Charles Flahault, BP 14491, 34093 Montpellier CEDEX 05, France; k.elcheikh@nanomedsyn.com

**Keywords:** porous silicon nanoparticle, surface functionalization, PEG, mannose, stealth properties, biodegradation kinetic, biocompatibility

## Abstract

A challenge regarding the design of nanocarriers for drug delivery is to prevent their recognition by the immune system. To improve the blood residence time and prevent their capture by organs, nanoparticles can be designed with stealth properties using polymeric coating. In this study, we focused on the influence of surface modification with polyethylene glycol and/or mannose on the stealth behavior of porous silicon nanoparticles (pSiNP, ~200 nm). In vivo biodistribution of pSiNPs formulations were evaluated in mice 5 h after intravenous injection. Results indicated that the distribution in the organs was surface functionalization-dependent. Pristine pSiNPs and PEGylated pSiNPs were distributed mainly in the liver and spleen, while mannose-functionalized pSiNPs escaped capture by the spleen, and had higher blood retention. The most efficient stealth behavior was observed with PEGylated pSiNPs anchored with mannose that were the most excreted in urine at 5 h. The biodegradation kinetics evaluated in vitro were in agreement with these in vivo observations. The biocompatibility of the pristine and functionalized pSiNPs was confirmed in vitro on human cell lines and in vivo by cytotoxic and systemic inflammation investigations, respectively. With their biocompatibility, biodegradability, and stealth properties, the pSiNPs functionalized with mannose and PEG show promising potential for biomedical applications.

## 1. Introduction

The emergence of nanomedicine has opened up opportunities for the development of more efficient anti-cancer agents that induce fewer side effects. Among these, porous Si-based nanoparticles (NPs) were found to be promising nanocarriers due to their biodegradability and biocompatibility [1,2,3,4,5,6]. Si-based NPs display unique features such as high specific surface area and large porous volume [5,7] that are particularly interesting for: (i) the imaging of cancerous tissues [8,9,10]; (ii) selective binding to receptors overexpressed by target tissues or cells type in conjugation with specific ligands or antibodies [11,12,13,14,15,16,17,18,19]; (iii) delivery of large concentrations of drugs and their controlled-release [1,20,21,22]; (iv) photosensitivity for photodynamic therapy (PDT) [23]; or (v) combination with two-photon excitation (TPE) in the near-infrared (IR) region that offers new perspectives for the treatment of solid tumors with an improved tissue penetration depth and spatial resolution [11,24]. 

One of the challenging issues concerning the development of such Si-based nanocarriers regards their recognition by the mononuclear phagocyte system (MPS) after their intravenous injection in the body. Such recognition will strongly decrease their performance and efficiency. With respect to NPs’ clearance by the MPS, the liver and the spleen are the most active organs because of phagocytic cells as macrophages that act by surface opsonization [25] and generally express similar membrane receptors to those of tumors [26]. Given other constraints, such as the heterogeneities of tumors in terms of enhanced permeability and retention (EPR) and the multifactor impact such as electrolytes, proteins and lipids onto colloidal stability of nanocarriers, less than 10% of the total administered drug dose reaches the tumor [2]. Based on these considerations, there is a need to develop stealth nanocarriers with reduced uptake by the MPS and optimized for the EPR effect.

One way to design NPs with such stealth properties is surface functionalization by polyethylene glycol (PEG) to increase the surface hydrophilicity, improve the circulation time, and decrease the immune response by preventing interactions with blood proteins and MPS cells [27,28]. In this regard, PEG-functionalization efficiency has already been reported in vitro [29] and in vivo [10,30]. Another promising approach to increase the local concentration of Si-based NPs in tumor tissue is to conjugate these NPs with targeting molecules that have a high affinity for tumor cells. Sugars have been the focus of several studies since their receptors (lectins) are overexpressed in most of the cancer cells [31]. One possible targeting agent is low biomolecular weight mannose (Man), which was successfully used to target human cancer cells, including retina, breast and prostate, due to its high specificity to lectin receptors [11,15,32,33,34]. Previous studies by Gary-Bobo et al. have shown that the immobilization of Man derivatives at the surface of porous SiO_2_ and Si NPs significantly increased the targeting of human breast (MCF-7, MDA-MB-231) and prostate (LNCaP) cancer cells by TPE–PDT therapy [11,15,32,33,34]. 

In this study, we designed porous silicon NPs (called pSiNPs) with PEG and/or Man functionalization in order to assess their stealth properties and biocompatibility in vivo after intravenous injection in mice. Biodistribution of pSiNP with PEG and mannose has not been studied yet to our knowledge. To quantify their avoidance by organs involved in the MPS and their clearance from the body, the Si content in target organs, blood and urine were measured using inductively coupled plasma mass spectrometry (ICP-MS). Complementary in vitro experiments were performed to assess the mechanisms of biodegradation and aggregation in standardized biological media. The chemical and colloidal stability of the formulations were compared by measuring the dissolution kinetics and aggregation states in the presence or not of fetal bovine serum (FBS). Finally, their biocompatibility was assessed in vivo by the plasma analysis of exposed mice and in vitro by measuring cell cytotoxicity. 

## 2. Results and Discussion 

### 2.1. Physico-Chemical Properties of Pristine and Formulated pSiNPs in Stock Suspensions

pSiNPs were synthesized by anodic etching of boron-doped crystalline silicon in a solution of aqueous hydrofluoric acid (HF) and ethanol, followed by electropolishing and fracturation of the porous layer. After centrifugation, NPs with a narrow-size distribution around 200 nm (using transmission electron microscopy (TEM)) were isolated. Oriented porosity with pore channels of 15–25 nm in diameter were observed, in agreement with average pore diameter calculated from the nitrogen adsorption/desorption analysis (19.4 nm). The pSiNPs also displayed a large surface area of 493 m^2^·g^−1^ (Figure S1).

The Bloch decay and cross-polarization magic angle spinning (CP-MAS) pulse sequences were used for the collection of ^29^Si nuclear magnetic resonance (NMR) (Figure S2). While Bloch decay collection gives information for all Si sites, CP-MAS is only sensitive to Si sites that are spatially close to protons i.e., the silanol groups at the surface of the NPs. In Bloch decay, only one peak was observed at approximately −110 ppm that was associated with siloxane and referred as Q4. In the ^29^Si CP-MAS spectrum, two spectral features at −8 and −90 ppm were observed. With a NPs size of ~200 nm, the percentage of surface atoms described by these features was estimated to be less than 2%. The contribution with a chemical shift centered around −90 ppm (90% of total signal) is associated with surface silicon atoms that are predominantly in the Q2 configuration (i.e., with 2 hydroxyl groups). The peak with a chemical shift of approximately −8 ppm could be attributed to C–Si and represented <0.2% of Si. Based on these NMR observations we concluded that, once dispersed in absolute ethanol, the majority of the Si atoms of the pSiNPs were bound to oxygen.

**Figure 1:**
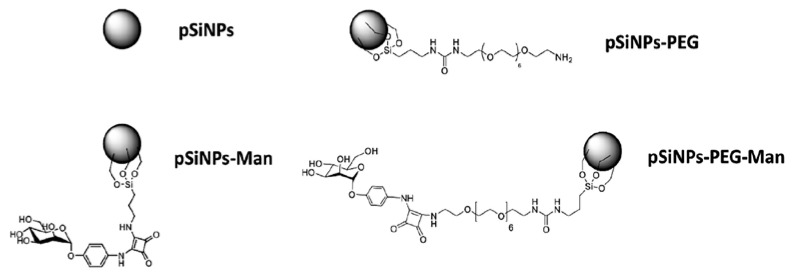
Schematic of the four pSiNPs formulations.

Besides pristine pSiNPs, three formulations were prepared containing PEG and/or Man (Figure 1). The grafting of the PEG involved the functionalization of PEG diamine moiety with ICPES (isocyanopropyltriethoxysilane) prior to covalent attachment to the pSiNPs by silanization reaction, with the silanol present at the surface of the NPs (Figure S3). The mannose could then attach to the pendant PEG chain by reaction of the phenyl squarate with the amine function of the PEG (Figure S4). Finally, the grafting of mannose alone also involved chemical modification of a mannose phenyl squarate with APTES (aminopropyltriethoxysilane) for further grafting to the pSiNPs by silanization reaction (Figure S5). The covalent attachment of PEG and Man at the surface was confirmed by attenuated total reflectance Fourier transform infrared spectroscopy (ATR-FTIR) (Figure S6). Infrared spectroscopy confirmed the oxidation of the pSiNPs which is favorable for a successful surface chemical modification and functionalization by silanization chemistry. The amount of grafted Man was determined spectrophotometrically by reaction with resorcinol [24], at 36 µg and 28 µg of mannose phenyl squarate per mg of pSiNPs for pSiNPs–Man and pSiNPs–PEG–Man, respectively (Figure S7). These values are comparable for both grafting methods.

In ethanol, these oxidized pristine pSiNPs had average hydrodynamic diameters centered on 200 ± 20 nm (vol. %) and 185 ± 18 (num. %) with a moderate polydispersity index (PDI) around 0.16 ± 0.02 consistent with TEM images, and a negative zeta potential (−25 ± 1 mV). This negative zeta potential is due to the oxidation of the NPs and is attributed to the silanol groups present at the surface of the pSiNPs. After functionalization by PEG and/or Man, an increase of the average hydrodynamic diameters was observed for pSiNPs-PEG to 315 ± 26 nm (vol. %) and 185 ± 18 nm (num. %), for pSiNPs–Man to 488 ± 103 nm (vol. %) and 404 ± 33 nm (num. %), and for pSiNPs–PEG–Man to 530 ± 56 nm (vol. %) and 528 ± 77 nm (num. **%**). Moderate PDI values (0.35 ± 0.2 and 0.22 ± 0.1) were observed for pSiNPs–PEG and pSiNPs–Man, while for pSiNPs–PEG–Man the PDI values were higher (0.68 ± 0.3). The size distribution of hydrodynamic diameters is provided in supporting information (Figure S8). Zeta potential measurements also confirmed the successful chemical functionalization of the NPs observed by ATR-FTIR (Table 1). Indeed, the functionalization with PEG induced a charge inversion from negative (−25 ± 1 mV) to positive (28 ± 2 mV) in ethanol. This highlights that the surface of the pSiNPs was successfully functionalized by the cationic aminated PEG molecules (Table 1). In comparison, modification of pSiNPs with Man slightly increased the zeta potential to −18 ± 1 mV in agreement with the successful functionalization of the surface by Man. Modifying the surface by both PEG and Man give a zeta potential value of 10 ± 0.2 mV showing the grafting of Man on PEG-functionalized pSiNPs. 

### 2.2. In Vitro Biodegradability Kinetics

**Figure 2:**
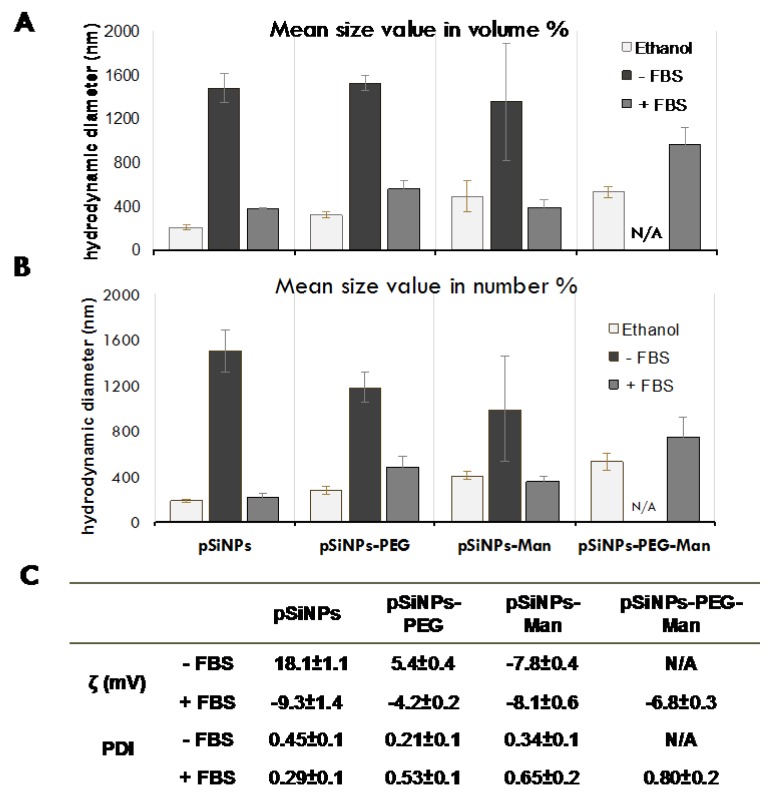
Average hydrodynamic diameters expressed as vol. % (**A**), num. % (**B**), and (**C**) ζ potential measurements and polydispersity index (PDI) for pristine or formulated pSiNPs after 10 min in DMEM/F12 ± 10% fetal bovine serum (FBS). The hydrodynamic diameters are mean size value (in vol. %). N/A denotes the absence of data due to strong aggregation. Data are the mean ± SD (*n* = 3).

**Figure 3:**
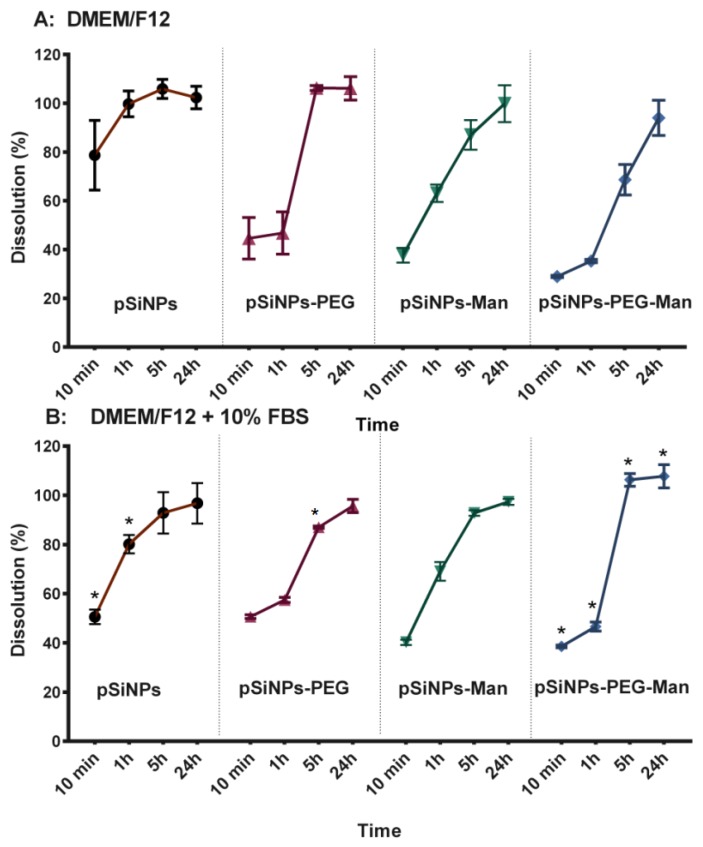
Dissolution kinetics of pSiNPs, pSiNPs–PEG, pSiNPs–Man, and pSiNPs–PEG–Man formulations after 10 min, 1, 5, and 24 h in DMEM/F12 ± 10% FBS. Results are expressed as a percentage of dissolved Si versus the total Si contents: (**A**) FBS-free DMEM/F12 medium; (**B**) 10% FBS-supplemented DMEM/F12 medium. Data are the mean ± SD (*n* = 3). Asterisk (*) denote statistical significant difference between dissolution rate in DMEM/F12 ± 10% FBS for pSiNPs formulations, * *p <* 0.05.

It is well known that the interactions between Si-based NPs and cancer cells depend not only on cell strains [5,35], but also on the size, surface properties, and solubility of the NPs in biological media [2,7,21,36,37]. Porous silicon has already been shown to be biodegradable in physiological environments, and to dissolve into orthosilicic acid, which is necessary for normal bone and connective tissue homeostasis [38]. Understanding the physical–chemical behavior of the pristine and formulated pSiNPs in standardized nutritive cellular media in vitro provided useful insights about their relative aggregation states and dissolution rates. The aggregation state (hydrodynamic diameter and zeta potential) and the dissolution rate were assessed in FBS-free and 10% FBS-supplemented Dulbecco’s modified Eagle’s medium DMEM/F12 (Figure 2 and Figure 3).

Aggregation state was first assessed 10 min after incubation in the FBS-free culture media. While well dispersed in their stock suspension, a strong aggregation occurred for all formulations. Aggregates with mean hydrodynamic diameters of around 1350–1520 nm (vol. %) and 1000–1500 nm (num. %) were quickly formed for pSiNPs, pSiNPs–PEG, and pSiNPs–Man. The pSiNPs–PEG–Man appeared to be the more destabilized (large and polydisperse aggregates), which makes them unmeasurable with dynamic light scattering (DLS). It is well established that in cell culture media, the neutral pH, the elevated ionic strength, and the presence of a divalent cation such as Ca^2+^ and Mg^2+^ can interact with the surface of the particles, decrease their surface charges, and cause their aggregation. This was also confirmed by zeta potential measurements in the FBS-free culture media (Figure 2B) that were closer to neutral in abiotic cell media at pH 7.4 (18.1 ± 1 mV for the pSiNPs, 5.4 ± 0.4 mV for pSiNPs–PEG, −7.8 ± 0.4 mV for pSiNPs–Man). This highlights the decrease of the electrostatic repulsion forces between NPs, which favored their colloidal destabilization [39,40] in standardized culture media.

As well as a colloidal instability, we also observed that formulated NPs were chemically unstable in the FBS-free DMEM/F12 medium. After 24 h, 100% of the Si from the four pSiNPs formulations were released as Si dissolved species. The dissolution kinetics were formulation-dependent. In FBS-free DMEM/F12 medium (Figure 3A), pristine pSiNPs shows the fastest degradation kinetic with 78 ± 14% of Si release in less than 10 min. Once functionalized with PEG and/or Man, Si mass loss from the NPs was reduced (~40% in 10 min). Indeed, after 1 h the percentages of dissolution were 99 ± 5% for pristine pSiNPs, 63 ± 4% for pSiNPs–Man, 46 ± 8% for pSiNPs–PEG, and 35 ± 1% for pSiNPs–PEG–Man. This highlights a chemical stabilization of the surface of the pSiNPs by the functionalization with organic molecules such as PEG and/or Man; the PEG–Man-functionalized NPs being the more stable in pure DMEM/F12 even after 5 h. 

Alongside formulation-dependency, the dissolution kinetics of the pristine pSiNPs were also serum-dependent (Figure 2 and Figure 3). Interaction with proteins is known to play a great role in the kinetics of biodegradation and cellular uptake [25,36,41]. Herein, in 10% FBS-supplemented media, the dissolution of pristine pSiNPs was significantly lower (50 ± 3%) than in FBS-free medium (78 ± 14%) after 10 min. This can likely be attributed to the adsorption of anionic proteins from the serum at the surface of pSiNPs [12,42,43,44]. These pSiNPs-protein interactions were confirmed by size and zeta-potential measurements. The addition of proteins in DMEM/F12-FBS media decreased the size of the pSiNPs aggregates and made their zeta potential negative (Figure 2). 

Similar decreases in the hydrodynamic diameters were observed for the pSiNPs–Man, pSiNPs–PEG, and pSiNPs–PEG–Man between the FBS-free DMEM/F12 and the DMEM/F12 complemented with 10% FBS. These aggregates had PDI values that were not statistically different compared to those measured in ethanol (large standard deviation) except for the pSiNPs–Man in the FBS-complemented DMEM/F12. Moreover, the zeta potential of these three formulations were all ranged between −4 and −8 mV in the FBS-complemented DMEM/F12. The comparable negative charge of these formulations suggested that protein adsorption dominated the surface charge distribution. However, such a protein adsorption did not impact the dissolution kinetics of pSiNPs–Man and pSiNPs–PEG. A similar release of Si was observed for the FBS-free and FBS-supplemented DMEM/F12, excepting pSiNP–PEG at 5 h (*p <* 0.05) (Figure 3B). PEG is known to be an efficient way to prevent non-specific protein adsorption at the surface of materials [45,46,47]. We hypothesized that, once formulated by PEG and Man, the Si atoms localized at the surface of the pSiNPs were less available to directly interact with proteins limiting their complexation–dissolution. Regarding pSiNPs–PEG–Man formulation, a significant difference in the dissolution kinetics was observed between the FBS-free and FBS-supplemented DMEM/F12 at all time points. The combined PEG–Man formulation dissolved faster in the presence of FBS, which shows that the combination had an effect on the accessibility of the Si atoms at the surface and their dissolution kinetics. 

### 2.3. Biocompatibility Studies In Vitro

**Figure 4:**
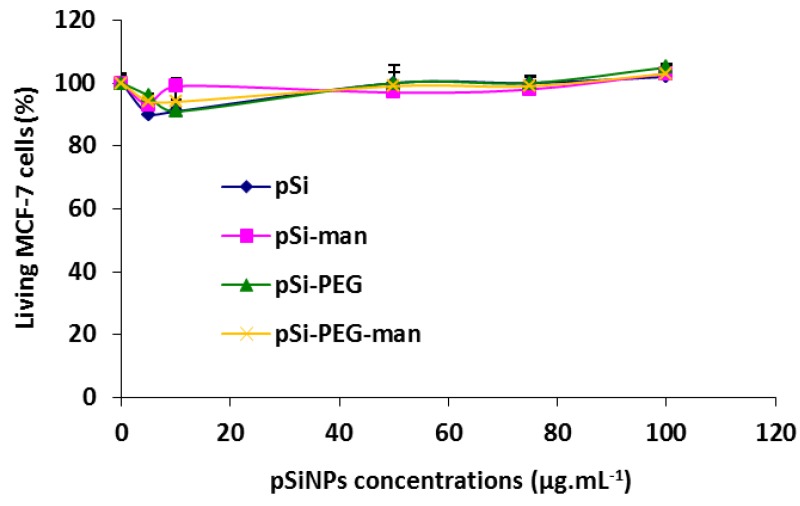
Cytotoxicity assessment of pSiNPs, pSiNPs–PEG, pSiNPs–Man, pSiNPs–PEG–Man. Human breast cancer cells (MCF-7) were incubated for 3 days with the 4 pSiNPs suspensions. Quantification of living cells was performed using (3-(4,5-dimethylthiazol-2-yl)-2,5-diphenyltetrazolium bromide (MTT) assay.

In order to assess the biocompatibility of these 4 pSiNPs formulations, we studied their effects on the cell growth of human breast cancer cells (MCF-7). Cells were incubated for 72 h with increasing concentrations of each formulation (from 5 to 100 mg·L^−1^). After three days, the number of living cells did not show any significant decrease compared to the control (Figure 4). Consequently, all the tested formulations did not induce any cell death or growth for the range of concentrations studied. This suggests an absence of cytotoxicity. 

### 2.4. Biodistribution and Clearance of the pSiNps Formulation In Vivo

**Figure 5:**
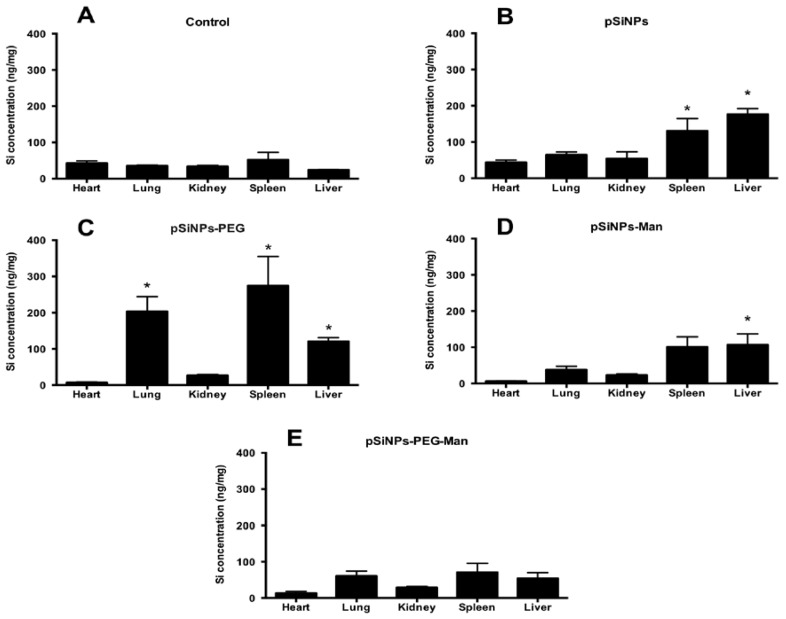
Si concentrations in organs of mice 5 h after intravenous injection. Control group (**A**), pSiNPs (**B**), pSiNPs–PEG (**C**), pSiNPs–Man (**D**) and pSiNPs–PEG–Man (**E**). Data are the mean ± SD (*n* = 3). Asterisk (*) denote statistical significant difference between pSiNPs-exposed mice and control group, * *p <* 0.05.

Based on the in vitro biodegradation result, the biodistribution of pristine and functionalized pSiNPs was assessed in vivo 5 h after intravenous injection into healthy mice. We observed that the Si biodistribution following injection of pSiNPs depended on the surface functionalization (Figure 5). As with other Si-based NPs [6,37,48], Si (dissolved or nanoparticulate forms) from pristine pSiNPs accumulated mainly in the MPS-related organs such as the liver and spleen (Figure 5B). This is likely due to pSiNPs being trapped by the Kupffer cells and splenic macrophages. Compared with pristine pSiNPs, pSiNPs functionalized with PEG induced less accumulation of Si in the liver. This suggests that the pSiNPs–PEG could partially escape from recognition and phagocytosis by liver phagocytes. Surprisingly, Si contents in the lung and spleen after pSiNPs–PEG treatment were higher than with pristine pSiNPs (*p <* 0.05) (Figure 5C), demonstrating that the PEGylated NPs could be captured by lung and splenic macrophages and hardly removed from the MPS organs. Faure et al. have shown the splenic capture of NH_2_-ended PEG grafted on hybrid nanoparticles, in agreement with a positive zeta potential [49].

On the contrary, functionalization with mannose significantly decreased the accumulation of Si in the organs. As shown in Figure 5D, only liver accumulation was significantly higher than the control group for pSiNPs–Man formulation (*p <* 0.05), likely due to the presence of Kupffer cells in this organ which express mannose receptors [50]. The best stealth effect was obtained for the pSiNPs–PEG–Man formulation. No significant accumulation of Si in the studied organs was observed for pSiNPs–PEG–Man (Figure 5E), indicating that PEGylation of pSiNPs and the post-functionalization with Man effectively reduced their uptake by the MPS. 

**Figure 6:**
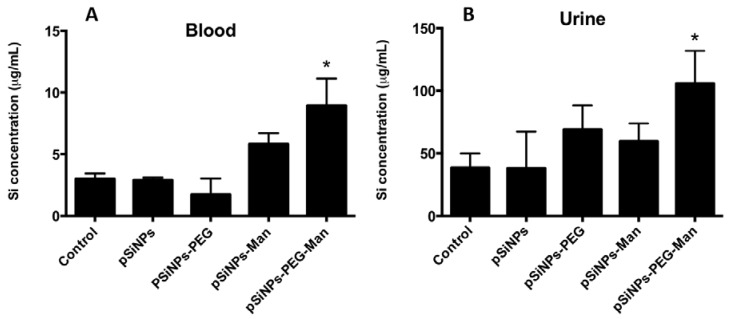
Si concentrations in blood (**A**) and urine (**B**) of mice after 5 h of intravenous injection. Mice were exposed to the four pSiNPs formulations. Data are the mean ± SD (*n* = 3). Asterisks (*) denote statistical significant difference between pSiNPs exposed mice and control group, *p <* 0.05.

The main objective of the surface modification of pSiNPs by PEG and Man was to escape from the MPS organs, increasing their blood-circulation lifetime and their excretion by urines. Figure 6 shows the Si concentration following injection of pristine pSiNPs, pSiNPs–PEG, pSiNPs–Man, and pSiNPs–PEG–Man in mice blood and urine 5 h after injection. For the pSiNPs, pSiNPs–PEG, and pSiNPs–Man, significant accumulation in the liver, spleen, and lung resulted in non-statistically different Si concentration in the blood and urine compared to the control group. However, for the pSiNPs–PEG–Man formulations, the absence of accumulation in the MPS organs is associated with a significant increase of Si concentration in the blood and urine compared to the control group (*p <* 0.05). In vivo, the mechanism of Si clearance is attributed to degradation of pSiNPs into soluble silicic acid followed by renal excretion. Such a biodegradation has been observed in vitro in the presence of FBS where a complete dissolution of the pSiNPs–PEG–Man was reached after 5 h. It is noteworthy that pSiNPs functionalized with PEG and Man was more destabilized in the FBS complementary DMEM/F12 medium. This indicates that differences in stealth behavior of the pSiNPs–PEG–Man formulation, compared to other three formulations in biological contexts, cannot be ascribed to a larger aggregate formation, but only to the differences in their surface functionalization. 

Both in vitro and in vivo experiments agreed on the fact that the PEGylated Man-modified pSiNPs are quickly biodegraded in the bloodstream allowing for their excretion by renal filtration in less than 5 h. By contrast, other studies have shown clearance of Si from the mice bodies following pSiNPs intravenous injection (130–180 nm) after one week [6]. Our results clearly evidence that PEG and Man protected pSiNPs from MPS system and prolonged their lifetime in blood circulation, and hence could enhance the dissolved Si excretion in urine. 

### 2.5. Biocompatibility Studies In Vivo

**Figure 7:**
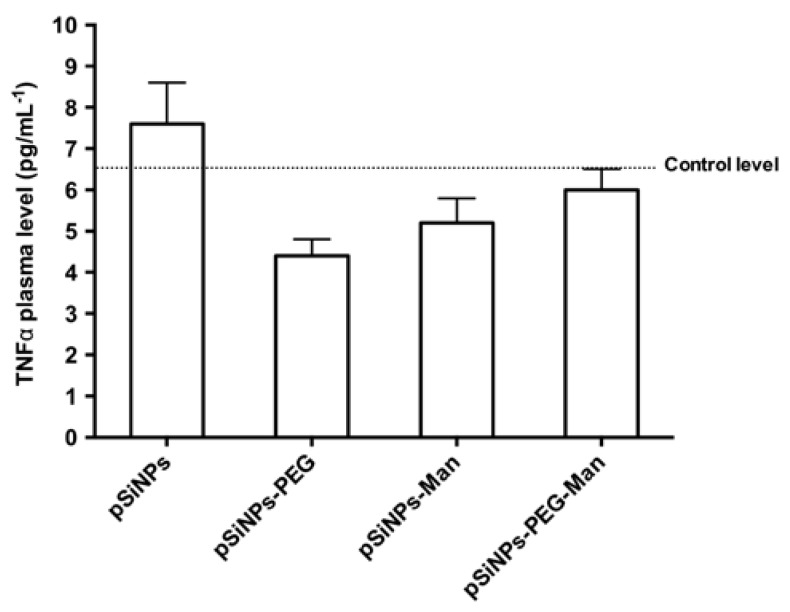
TNFα plasma level of mice treated intravenously by pSiNPs, pSiNPs–PEG, pSiNPs–Man, pSiNPs–PEG–Man at the final concentration of 40 mg·kg^−1^. Plasma were collected during the sacrifice, 5 h after treatment. Values are means ± standard deviation.

At the sacrifice, the blood of the mice was collected and plasma were prepared. In order to mesure the level of TNFα. Figure 7 shows that no significant increase in TNFα plasma level was observed in animals treated with each formulation. This suggests an absence of systemic inflammation at least within 5 h after injection.

## 3. Materials and Methods 

### 3.1. pSiNPs Synthesis

Boron-doped p^++^-type Si (0.8–1.2 mΩ.cm resistivity, <100> orientation) from Siltronix (France) was electrochemically etched in a 3:1 (*v*:*v*) solution of aqueous 48% hydrofluoric acid (HF): absolute ethanol (Sigma-Aldrich, Saint Quentin Fallavier, France). Etching was performed in a Teflon cell with a platinum ring counter electrode. A constant current of 167 mA·cm^−2^ was applied for 150 s, and then the sample was rinsed three times with ethanol. The porous layer was then removed from the substrate by application of a constant current of 4 mA·cm^−2^ for 250 s in an electrolyte solution containing 1:20 (*v*:*v*) aqueous 48% hydrofluoric acid: absolute ethanol. After rinsing three times with ethanol, the porous layer was put in ethanol in a glass vial. After 20 min of degassing under a nitrogen stream, the porous silicon film was fractured by ultrasonication for 16 h. The largest particles were then removed by spinning them down by centrifugation at 2700× *g* for 2 min (Minispin, Eppendorf, AG, Hamburg, Germany). In order to remove the smallest particles, the solution was finally centrifuged at 22,000× *g* for 30 min (centrifuge Eppendorf 5804, AG, Hamburg, Germany). The pellet was then dispersed in absolute ethanol.

### 3.2. pSiNPs Functionalization With PEG

0.5 mmol of polyethylglycoldiamine (368.5 g·mol^−1^) and 0.25 mmol of isocyanopropyltriethoxysilane (ICPES) were added with 2 mL of dried tetrahydrofuran (THF) in a 25 mL round-bottomed flask. The mixture was stirred overnight at 50 °C and under nitrogen. Freshly prepared pSiNPs were centrifuged at 22,000× *g* for 30 min in ethanol, then centrifuged once with THF. 30 mg of pSiNPs were dispersed in 2 mL of THF with ICPES-PEG and reacted overnight at 50 °C under nitrogen. After the reactions, the pSiNPs–PEG were centrifuged at 22,000× *g* and washed 5 times with absolute ethanol to remove the PEGdiamine physisorbed onto their surface. 20 mg of pSiNPs–PEG were finally dispersed in 10 mL of absolute ethanol.

### 3.3. pSiNPs–PEG Functionalization With Man

0.03 mmol of mannose (*p*-[*N*-(2-Ethoxy-3,4-dioxocyclobut-1-enyl)amino]phenyl-α-d-mannopyranoside) (395.6 g·mol^−1^), 0.07 mmol of trimethylamine, and 30 mg of pSiNPs–PEG were added with 2 mL of DMF in a 25 mL round-bottomed flask. The mixture was stirred overnight at 60 °C under nitrogen. After the reactions, the pSiNPs–PEG–Man were centrifuged at 22,000× *g* and washed 5 times with absolute ethanol to remove the mannose physisorbed onto their surface. 20 mg of pSiNPs–PEG–Man were finally dispersed in 10 mL of absolute ethanol.

### 3.4. pSiNPs Functionalization With Man

0.03 mmol of mannose (*p*-[*N*-(2-Ethoxy-3,4-dioxocyclobut-1-enyl)amino]phenyl-α-d-mannopyranoside) (395.6 g·mol^−1^), 0.04 mmol of aminopropyltriethoxysilane (APTES), and 11 µL (0.07 mmol) of triethylamine were added in 2 mL of dried THF in a 25 mL round-bottomed flask,. The mixture was stirred overnight at room temperature and under nitrogen. Freshly prepared pSiNPs were centrifuged at 22,000× *g* for 30 min in ethanol, then centrifuged once with DMF. 30 mg of pSiNPs were dispersed in 3 mL of DMF with APTES-Man and reacted overnight at 60 °C under nitrogen. After the reactions, the pSiNPs–Man were centrifuged at 22,000× *g* and washed 5 times with absolute ethanol to remove the mannose physisorbed onto their surface. 20 mg of pSiNPs–Man were finally dispersed in 10 mL of absolute ethanol.

### 3.5. Characterizations of pSiNPs Initial Suspensions in Ethanol

Solid-state ^29^Si nuclear magnetic resonance (NMR) was used to assess the Si speciation of pSiNPs before functionalization by PEG and Man, or in vivo/in vitro studies. NMR spectra were obtained on a Bruker Advance 400 WB spectrometer ( Bruker BioSpin Corporation, Billerica, ME, USA) at 79.5 MHz. Spectra were acquired in both single pulse and solid-state cross polarization magic angle spinning (CP-MAS) modes. In both cases the spin rate was 10 kHz and the number of scans was 4096. Recycle delays were 20 s and 5 s for the single pulse and the CP-MAS experiments respectively. Free induction decay (FID) was processed using the MestRecNova software (Mestrelab Research, Santiago de Compostela, Spain). A 50 Hz line broadening was applied prior to the Fourier transform, and phase and baseline correction were done manually. The relative proportion of the surface Si species was calculated from peak integration.

TEM images of the NPs were obtained on the JEOL 1200 EXII instrument (Jeol Ltd., Tokyo, Japan). The specific surface area and pore-size distribution of the samples were estimated using nitrogen adsorption–desorption isotherms with a Micrometrics ASAP 2020 instrument (ASAP 2020, Micromeritics Inc., Norcross, GA, USA). Before the sorption measurement, the samples were degassed at 80 °C for 6 h under reduced pressure. The specific surface area of the sample (*S*_BET_) was calculated according to the Brunauer, Emmett, Teller (BET) method from the linear part of the nitrogen adsorption isotherm, and the pore-size distribution were analyzed according to the Barrett, Joyner and Halenda (BJH) method. Infrared spectra were recorded on a Nicolet IS5 spectrometer (Nicolet Instrument, Thermo Company, Waltham, MA, USA) with the attenuated total reflectance (ATR) ID5 module to study the covalent attachement of PEG and Man at the surface of the pSiNPs.

Hydrodynamic diameters and zeta potentials of the initial and functionalized NPs were measured in absolute ethanol by DLS using the NanoZS (Malvern Instruments Ltd., Worcestershire, UK) and the zeta potential measurements using the Zetasizer (Malvern Instruments Ltd., Worcestershire, UK). For data acquisition, a refractive index (RI) of 1.54 was used, and the reading was carried out at 173° with respect to the incident beam. 

### 3.6. Dosing of the Phenyl Squarate Mannose Grafted on the pSiNPs–Man and pSiNPs–PEG–Man Formulations

The amounts of phenyl squarate-mannose grafted on the pSiNPs were determined by specific colorimetric reaction using a lambda 35 de Perkin Elmer UV–Vis spectrometer. The molar absorption coefficient of the mannose was determined using a solution of mannose in hydrated sulfuric acid and resorcinol (1,3-dihydroxybenzene) at different concentrations. The mannose amounts ranging from 1 to 4 mg were dissolved in 20 mL of acetic acid (0.01 mol·L^−1^). Then, 200 µL of each solution was added to the solution containing 1 mL of 75% sulfuric acid and 200 µL of resorcinol. The samples were vortexed for 5 min and heated at 90 °C for 30 min. The samples were placed in the fridge and in darkness for 30 min. The absorbance of the solution was recorded between 350 and 600 nm. Calibration curves were also recorded, in order to determine the molar absorption coefficient of the mannose under the same conditions (ε_422_ = 11651 L·mol^−1^·cm^−1^). The 75% sulfuric acid (1 mL), resorcinol (200 µL) and acetic acid (200 µL) solution was used as a reference solution. The dosing of the mannose grafted onto the pSiNPs followed the procedure described above. Here, the mannose solution was replaced using a known quantity of NPs functionalized with mannose phenyl squarate. A loading of 36 µg and 27.6 µg of mannose phenyl squarate per mg of pSiNPs was obtained for pSiNPs–Man and pSiNPs–PEG–Man respectively.

### 3.7. Physico-Chemical Stability In Vitro

The colloidal stability of the pSiNPs, pSiNPs–PEG, pSiNPs–Man, pSiNPs–PEG–Man was studied in vitro in abiotic cell culture media. Before use, each formulation was sonicated in a water bath for 5 min. The NPs were then diluted at 40 mg·L^−1^ in Dulbecco’s modified Eagle’s medium supplemented with Glutamax (DMEM/F-12) with and without 10% FBS and 1% penicillin-streptomycin (Gibco Laboratories, Grand Island, NY, USA). The samples were stirred (100 rpm) at a constant temperature of 37 °C for 10 min, 1 h, 5 h and 24 h. At each selected time, 2 mL of mixing solution was used for DLS and zeta potential measurements (using NanoZS and Zetasizer, Malvern Instruments Ltd., Worcestershire, UK).

The chemical stability (release of dissolved species) of the pSiNPs, pSiNPs–PEG, pSiNPs–Man, pSiNPs–PEG–Man was studied in vitro in abiotic DMEM/F-12 medium with and without 10% FBS. After 10 min, 1 h, 5 h and 24 h, aliquots (4 mL) of stirred (100 rpm) suspensions were ultrafiltrated using centrifugal filter units (Amicon ultra4, 3 kDa molar mass membrane cutoff, Beckman Coulter, Inc., Fullerton, CA, USA). The collected fractions were diluted 10-fold in ultrapure water with 2% nitric acid before ICP-MS analysis (Thermo X series II model equipped with a collision cell, Fisher Corp., MA, USA). To avoid the loss of Si on filter membranes, the recovery of soluble silicon was tested and found to be close to 100%. Concentrations of Si in stock suspensions were also measured by ICP-MS by transferring 2 mL of suspension into 1 mL of HCl (35%) and 1 mL of HNO_3_ (67%) for NPs, followed by 45 min of microwave digestion. Samples were left in acid overnight for the digestion to complete. Appropriate dilutions were made for ICP-MS analysis. Results of the ICP-MS measurement are presented as means (*n* = 3) and standard deviations (SD).

### 3.8. In Vitro Cytotoxicity on Cell Line

Human breast cancer cells (MCF-7) were purchased from ATCC (American Type Culture Collection, Manassas, VA, USA). Cells were cultured in DMEM-F12 supplemented with 10% FBS and 50 µg·mL^−1^ gentamycin. These cells were allowed to grow in a humidified atmosphere at 37 °C under 5% CO_2_. To study the effects of NPs on cell growth, MCF-7 cells were seeded into 96-well plates at 2000 cells per well in 200 µL culture medium and allowed to grow for 24 h. Increasing concentrations of pSiNPs, pSiNPs–PEG, pSiNPs–Man, pSiNPs–PEG–Man were incubated in a culture medium of MCF-7 cells during 72 h. Then, a MTT assay was performed to evaluate the toxicity. Briefly, cells were incubated for 4 h with 0.5 mg·mL^−1^ of MTT (3-(4,5-dimethylthiazol-2-yl)-2,5-diphenyltetrazolium bromide; Promega, Madison, WI, USA) in media. The MTT/media solution was then removed and the precipitated crystals were dissolved in EtOH/DMSO (1:1). The solution absorbance was read at 540 nm.

### 3.9. Biodistribution in Mice

Female C57/BL6 mice aged 8 weeks were provided by Charles River and, housed in the institutional animal house under standard environmental conditions (23 ± 1 °C, 55 ± 5% humidity and 12 h/12 h light/dark cycles) and maintained with free access to standard diet and water. All animals (3 mice per condition) were intravenously exposed to the four pSiNPs formulations (pSiNPs, pSiNPs–PEG, pSiNPs–Man, pSiNPs–PEG–Man) at the final concentration of 40 mg·kg^−1^. Control mice were injected with the vehicle alone (200 µL of physiological serum). The mice were sacrificed 5 h after intravenous injection. Their blood, urine and major organs (heart, lung, spleen, kidney and liver) were collected. Si concentration was then quantified by ICP-MS. 

For quantification of the Si concentration in each organ (lung, kidney, spleen, heart and liver) of the exposed or control mice, the organs were freeze-dried, crushed and homogenized. Then, 1 mL of HNO_3_ (67%) and 1 mL of H_2_O_2_ were added to approximately 30–50 mg of organ powder. For quantification of Si in bio fluids, 100 μL of blood and 5 to 100 μL of urine were separately mixed with 1 mL of HNO_3_ (67%) and 1 mL of H_2_O_2_. The samples were left in acid overnight for digestion, and this was followed by 45 min of microwave digestion. Then, all the samples were diluted in HNO_3_ (2%) to reach a final volume of 10 mL, and Si concentrations were measured by ICP-MS. The ICP-MS measurement data are presented as means (*n*= 3 mice per condition) and standard deviations (SD). The data were analyzed using a two-way analysis of variance (ANOVA) test followed by a Sidak’s multiple comparisons test (Graphpad Prism 6, Graphpad Sofeware Inc., San Diego, CA, USA). A level of *p <* 0.05 was considered significant. 

### 3.10. TNFα Plasma Levels Measurement

A blood sample for each mouse was collected to determine the level of inflammatory biomarkers such as TNFα. Plasma was separated from the blood by centrifugation (15 min, 1200× *g*, 4 °C). Plasma TNFα levels were quantified using commercial enzyme-linked immunosorbent assay (ELISA) kits as described in the manufacturer’s protocol (R&D systems, Minneapolis, MN, USA).

## 4. Conclusions

The aim of this study was to design stealth pSiNPs using biodegradable pSiNPs and new surface-grafting with PEG and Man. When the PEGylated–pSiNPs were functionalized with Man, the pSiNPs–PEG–Man system exhibited a remarkable stealth property, with higher blood retention and a significant clearance in renal excretion. By contrast with studies reporting the influence of PEG on biodistribution [37,51], we observed that PEG at 368.5 g·mol^−1^ molecular weight had no significant effect on combined uptakes in major MPS organs such as the liver and spleen. Moreover, in vitro biodegradation kinetic studies were in accordance with in vivo studies indicating a fast biodegradation kinetic (~100% at 5 h in DMEM/F12-FBS medium). Based on the in vitro and in vivo biodegradation, biodistribution, clearance, and biocompatibility evaluations presented, the pSiNPs functionalized with Man and PEG-Man deserve to be tested in vivo to specifically target cancer sites in a non-invasive manner.

## Figures and Tables

**Table 1:** Zeta potential and hydrodynamic diameters (mean size value in vol. % and num. %) and PDI (polydispersity index) of pristine and functionalized pSiNPs in absolute ethanol. Data are the mean ± SD (*n* = 3).

Nanoparticles	pSiNPs	pSiNPs–PEG	pSiNPs–Man	pSiNPs–PEG–Man
Zeta potential (mV)	−25 ± 1	28 ± 2	−18 ± 1	10 ± 0.2
Hydrodynamic diameter (nm) vol. %	200 ± 20	315 ± 26	488 ± 103	530 ± 56
Hydrodynamic diameter (nm) num. %	185 ± 18	276 ± 35	404 ± 33	528 ± 77
PDI	0.16 ± 0.02	0.35 ± 0.2	0.22 ± 0.1	0.68 ± 0.3

## Supplementary Materials

The following are available online at http://www.mdpi.com/2079-4991/7/10/288/s1, Figure S1: Characterizations of the pristine pSiNPs. (A) Transmission electron microscopy (TEM) images, (B) N_2_ adsorption/desorption isotherm, Figure S2: CP-MAS spectra (black line) and Bloch decay spectra (red line) of ^29^Si NMR of pSiNPs suspended in absolute ethanol, Figure S3: Reaction pathway for the covalent binding of PEG on pSiNPs, Figure S4: Synthesis scheme for the functionalization of pSiNPs–PEG with Mannose, Figure S5: Reaction pathway for the covalent binding of mannose on pSiNPs, Figure S6: (A) DRIFT spectra of bare pSiNPs (brown), pSiNPs functionalized with PEG (blue) and pSiNP-PEG-Man (green), (B) DRIFT spectra of bare pSiNPs (brown), pSiNPs functionalized with Mannose phenyl squarate pSiNP-Man (green), Figure S7: UV-vis spectrum for quantification of mannose phenyl squarate in (A) pSiNPs–Man and (B) pSiNPs–PEG-Man, Figure S8: Size distribution curves in vol. % and num. % of four pSiNPs formulations in absolute ethanol.
