# Supplementary material for: Stealth Biocompatible Si-Based Nanoparticles for Biomedical Applications
Source: Nanomaterials (Basel). 2017 Sep 23;7(10):288. doi: 10.3390/nano7100288 (PMC5666453; doi:10.3390/nano7100288)
Supplement: Supplementary file 1 [file nanomaterials-07-00288-s001.pdf]

Supplementary Nanomaterials:

Stealth Biocompatible Si-Based Nanoparticles for Biomedical Applications

Wei Liu [1], Arnaud Chaix [2], Magali Gary-Bobo [3], Bernard Angeletti [1], Armand Masion [1], Afitz Da Silva [3,4], Morgane Daurat [3,4], Laure Lichon [3], Marcel Garcia [3], Alain Morère [3], Khaled El Cheikh [4], Jean-Olivier Durand [2], Frédérique Cunin [2] and Mélanie Auffan [1,*]

I. Characterization of pSiNPs initial suspensions in ethanol.

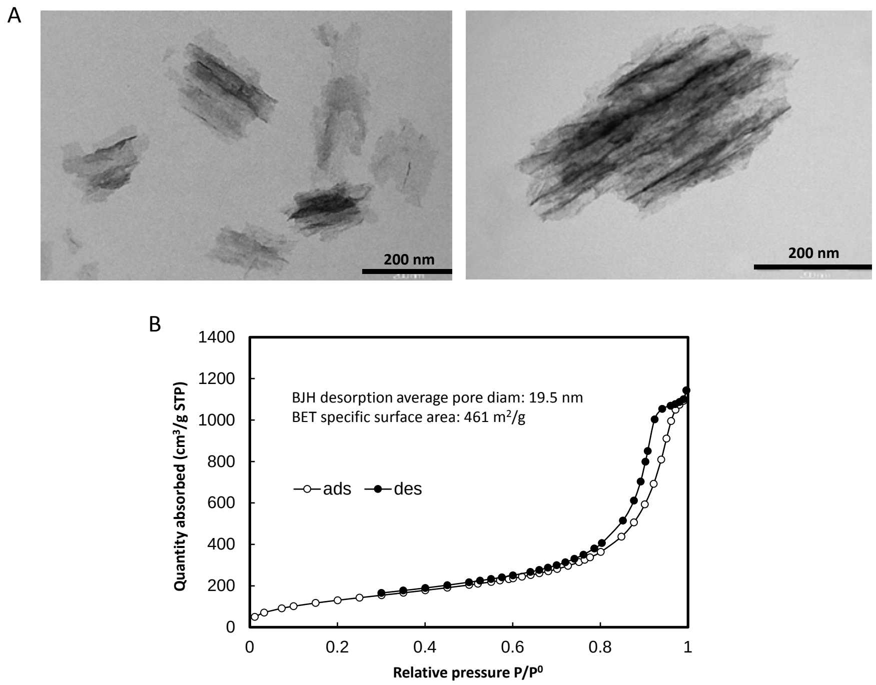

Figure S1. Characterizations of the pristine pSiNPs. (**A**) Transmission electron microscopy (TEM) images, (**B**) N_2 adsorption/desorption isotherm.

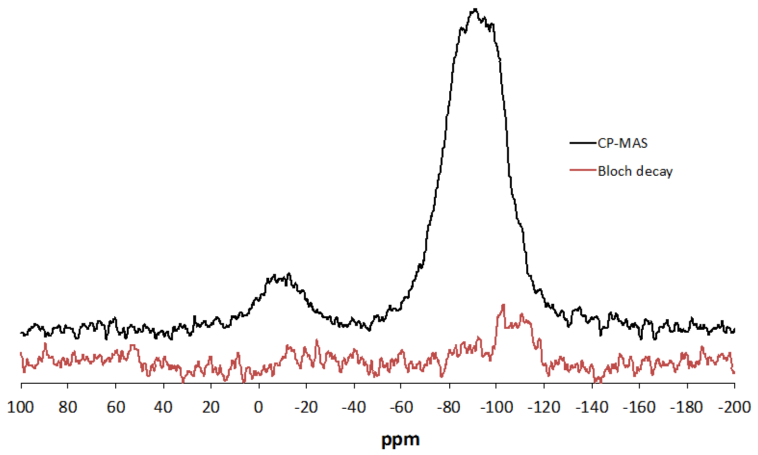

Figure S2. CP-MAS spectra (black line) and Bloch decay spectra (red line) of ^{29}Si NMR of pSiNPs suspended in absolute ethanol.

II. Surface functionalization of the pSiNPs

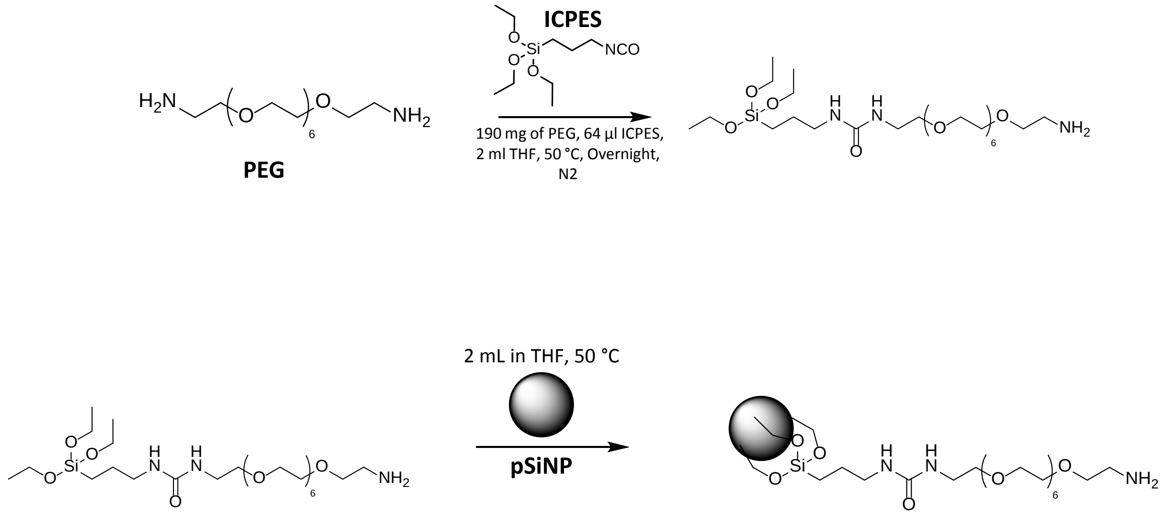

Figure S3. Reaction pathway for the covalent binding of PEG on pSiNPs.

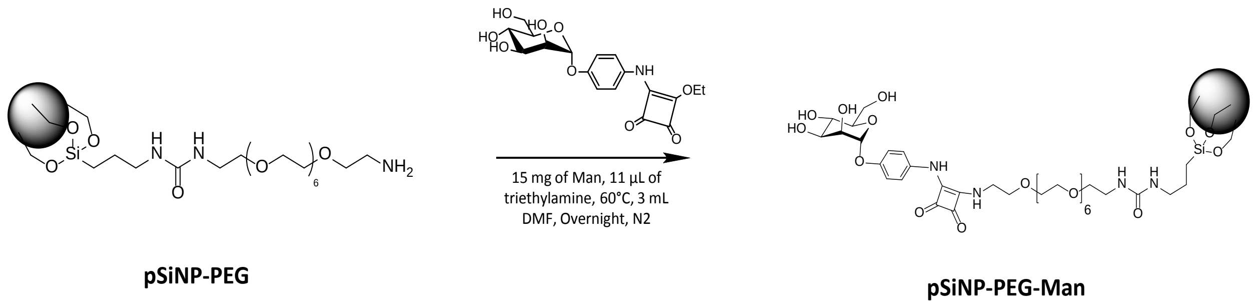

Figure S4. Synthesis scheme for the functionalization of pSiNPs–PEG with Mannose.

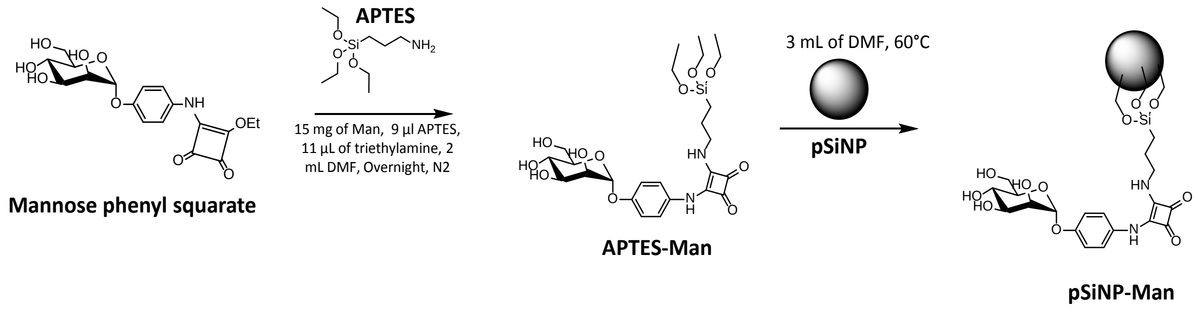

Figure S5. Reaction pathway for the covalent binding of mannose on pSiNPs.

III. Characterization of the functionalized pSiNPs

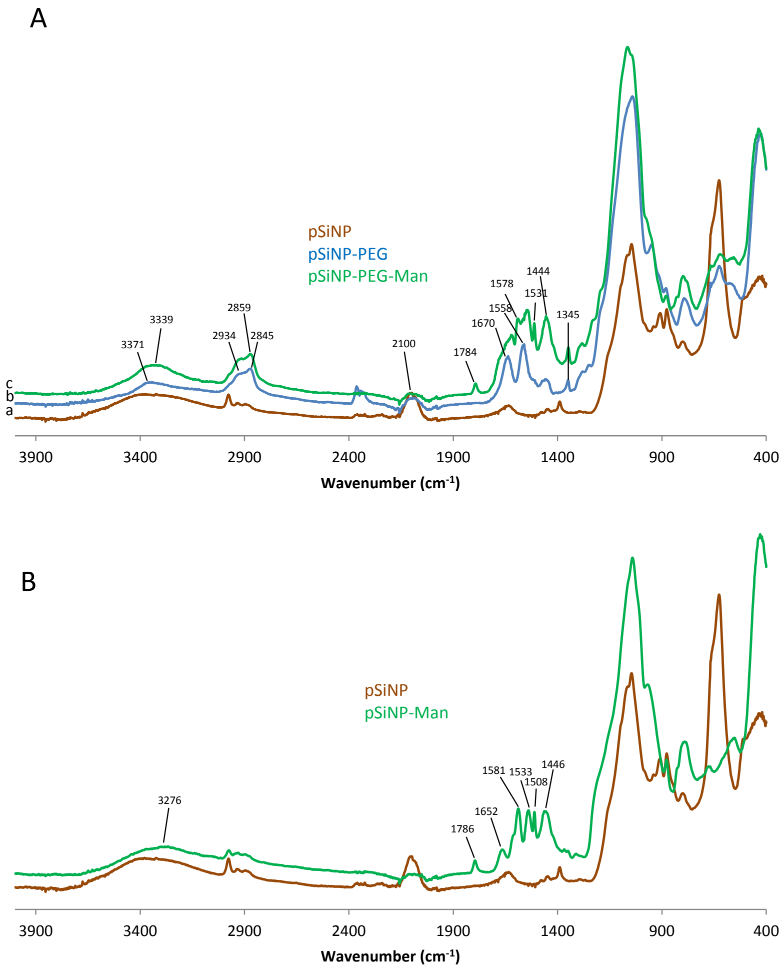

Figure S6. (**A**) DRIFT spectra of bare pSiNPs (brown), pSiNPs functionalized with PEG (blue) and pSiNP–PEG–Man (green), (**B**) DRIFT spectra of bare pSiNPs (brown), pSiNPs functionalized with Mannose phenyl squarate pSiNP–Man (green).

The band at 2100 cm^{-1} corresponds to the Si-H stretching vibration of the pSiNPs (Figure S6A). After the silanisation of the pSiNP with the APTES–PEG, the intense band observed at 2845-2934 cm^{-1} corresponds to the stretching vibration of the C-H in the PEG chain (Figure S6A). Additionally, the intense band observed at 1670 cm^{-1} corresponds to the thiourea group indicating the covalent attachment of the APTES–PEG on the pSiNPs. Finally, the band observed at 1558 cm^{-1} corresponds to the angular deformation of the primary N–H bond (Figure S6A). After the functionalization with the mannose (Figure S6A and B) the vibrational spectra appears complex with different bands between 1400 cm^{-1} and 1600 cm^{-1}, which can be assigned to the C=C bond of the phenyl and of the squarate linker of the mannose. Moreover, the presence of a large band centered at 3200-3400 cm^{-1} is attributed o ν(O-H) stretching vibration mode from the mannose.

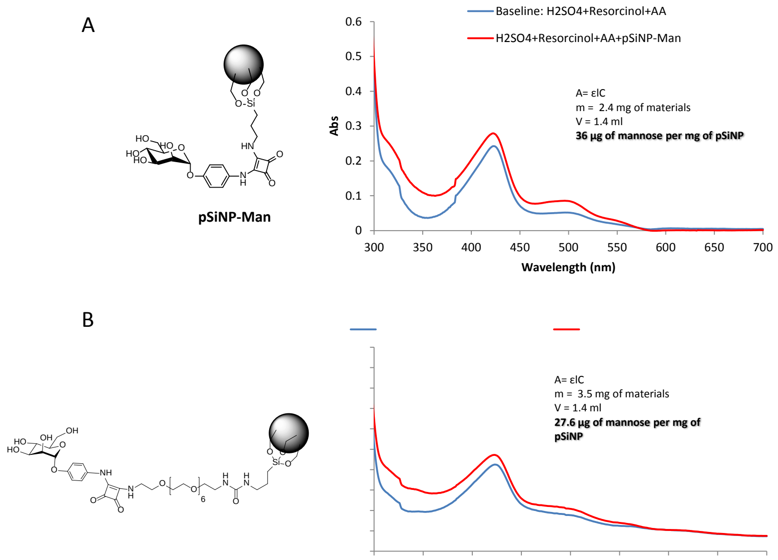

Figure S7. UV-vis spectrum for quantification of mannose phenyl squarate in (**A**) pSiNPs–Man and (**B**) pSiNPs–PEG–Man.

IV. Hydrodynamic diameter distribution of the functionalized pSiNPs

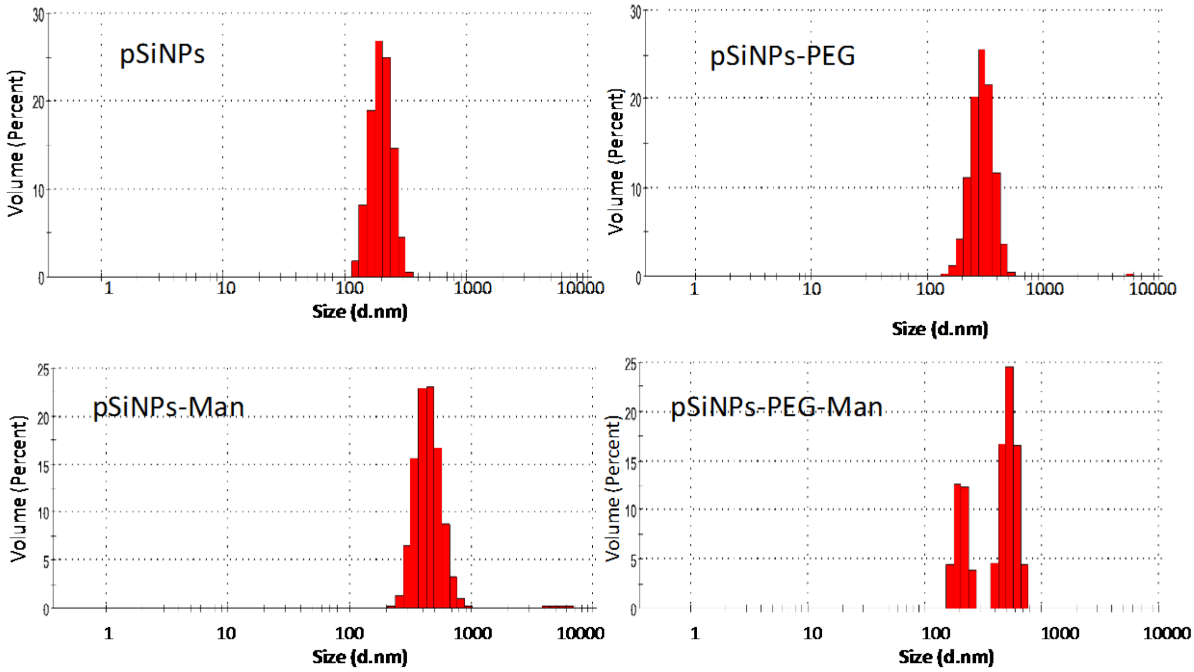

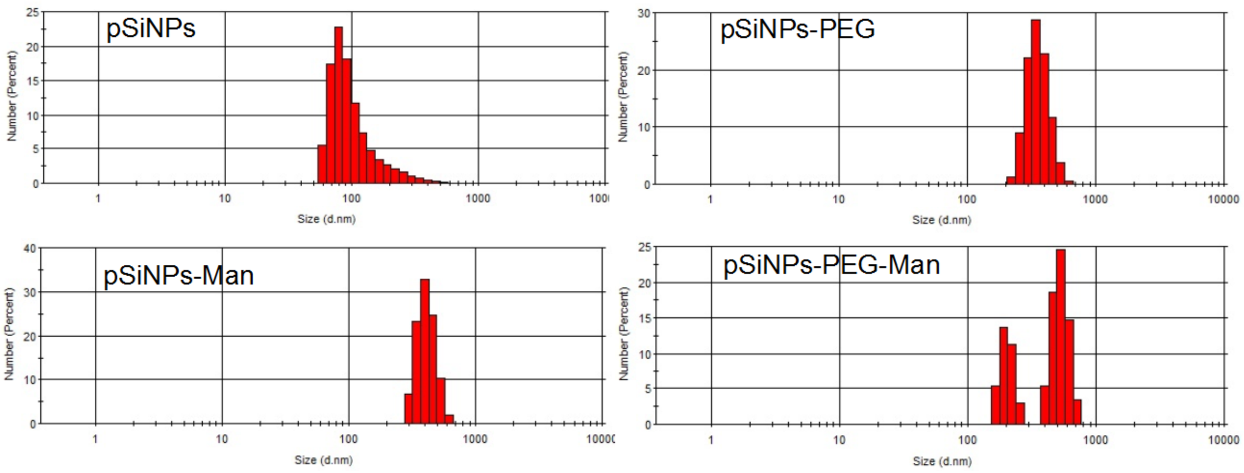

Figure S8. Size distribution curves in volume % (top) and in number % (bottom) of the four pSiNPs formulations in absolute ethanol.
